# Supplementary material for: Machine learning based energy-free structure predictions of molecules, transition states, and solids
Source: Nat Commun. 2021 Jul 22;12:4468. doi: 10.1038/s41467-021-24525-7 (PMC8298673; doi:10.1038/s41467-021-24525-7)
Supplement: Supplementary file 1 — Supplementary Information [file 41467_2021_24525_MOESM1_ESM.pdf]

Machine learning based energy-free structure predictions of molecules (closed and open-shell), transition states, and solids
Supplementary Material

Dominik Lemm[1], Guido Falk von Rudorff[1] and O. Anatole von Lilienfeld[1,2]
[1]*Faculty of Physics, University of Vienna, Kolingasse 14, 1090 Wien, Austria*
[2]*Institute of Physical Chemistry and National Center for Computational Design and Discovery of Novel Materials (MARVEL), Department of Chemistry, University of Basel, 4056 Basel, Switzerland*
*Electronic address: anatole.vonlilienfeld@univie.ac.at
(Dated: 16 June 2021)

I. SUPPLEMENTARY METHODS

A. Bond Length based Representations

To construct representations from a molecular graph that rely on bond lengths, covalent atomic radii are required for each type of bond (single, double, triple). Using the atomic radii as weights, the bond length distance or shortest path l_{ij} between two atoms i and j in a graph is calculated using Dijkstra's algorithm as implemented in igraph. Calculating the bond length distances for all atom pairs in a molecule results in a representation of the following form:

$$\text{Bond Length}_{ij} = \begin{cases} 0, & i = j, \\ l_{ij}, & i \neq j. \end{cases} \tag{1}$$

with l_{ij} being the bond length distance/shortest path between the atoms i and j. To include more physics, the bond length distance can be used to approximate 2-body interactions that are commonly used in QML representations such as the Coulomb Matrix (CM) or Bag-of-Bonds (BoB). The CM representation contains the coulomb interaction scaled by the interatomic distance as off-diagonal elements, while the diagonal represents an approximation to the atomic energy of the nuclear charge Z_i. This leads to a representation with the following form:

$$\text{CM}_{ij} = \begin{cases} 0.5Z_i^{2.4}, & i = j, \\ \frac{Z_i Z_j}{|\boldsymbol{R}_i - \boldsymbol{R}_j|}, & i \neq j. \end{cases} \tag{2}$$

with nuclear charge Z and atomic coordinates $\boldsymbol{R}$. Since the atomic coordinates are not available for a structure prediction task, the representation has to be adapted for molecular graphs. The bond length distance approach described above suits as an approximation to the intermolecular distance and can therefore be used to adapt the off-diagonal term of the CM to work in a graph setting. The adapted representation, dubbed graph CM, has the following form:

$$\text{graph CM}_{ij} = \begin{cases} 0.5Z_i^{2.4}, & i = j, \\ \frac{Z_i Z_j}{l_{ij}}, & i \neq j. \end{cases} \tag{3}$$

with nuclear charge Z and bond length distance l_{ij}. To convert the CM into a BoB representation, the CM has to be vectorized by grouping all matrix terms into specific bins. The thereby created canonical order (bag of bonds) ensures that during the kernel calculation only similar bins are compared. Each bin describes a particular bond type (H-H, C-C, C-H etc.). In this regard, the BoB and graph BoB representation use the same components as their respective matrix counterpart (CM and graph CM), but only differ through transforming the matrix into a canonical vector. Since the distance matrix is sorted based on the sorting of the representation, the distance matrix undergoes the same vectorization and binning procedure as the graph BoB representation.

B. Z-Matrix Learning

A alternative internal coordinate representation of atomistic structures is the so called Z-matrix. Instead of using pairwise distances, a Z-matrix contains information about bond distances, bond angles as well as dihedral angles. Conversion between a Z-matrix and Cartesian coordinates is possible. G2s has been used to predict bond distances, bond angles and dihedral angles,

respectively. Contrary to the distance matrix approach, the sorting of the representation was only dependent on the atom indices of the respective Z-matrix entry, making the machines independent of how the Z-matrix has been constructed. While bond distances and angles appear easier to learn, achieving remarkable accuracies of 0.01 Å MAE on distances and 2 degree MAE on bond angles, the learning of dihedral angles only achieved a MAE of 36 degree. The conversion from Z-matrix to a reasonable 3D geometry was not possible given these errors. It is worth to mention that the Z-matrix conversion suffers from error propagation, amplifying errors from atom to atom during the reconstruction. The distance geometry problem is superior in this regard since the compatibility of all distances is being optimized, leading to error cancellation instead of propagation.

C. Software

The Graph To Structure software is build upon Numpy[1], Scipy[2], Quadpy[3], RDKit[4] and igraph[5]. To extract adjacency matrices from xyz-files, the xyz2mol[6,7] package has been used. For high performance kernel ridge regression, the QML[8] package was used. Visualizations have been created using Matplotlib[9], Seaborn[10] and VMD[11].

II. SUPPLEMENTARY TABLES

Supplementary Table I. Baseline and test errors of structure generation methods. Errors are reported in terms of mean MAE of pairwise distances and RMSD for structures with (w) and without (w/o) hydrogen atoms, respectively. For the machine learning methods, the results of the largest training set size have been reported. 1) Errors towards a reference geometry, when the reference geometry has been optimized with one of the listed methods (UFF, MMFF, GFN2-xTB, PM6). 2) Structure generation with ETKDG from RDKit. Rows with UFF/MMFF have subsequently been optimized with either force field. 3) Structure generation with Gen3D from Open Babel with and without force field optimization. 4) Structure generation with G2S using the listed representations. 5) Hydrogen prediction with G2S.

		$C_7O_2H_{10}$				C_7NOH_{11}				E2/S_N2 Reactants			
		MAE [Å]		RMSD [Å]		MAE [Å]		RMSD [Å]		MAE [Å]		RMSD [Å]	
		w	w/o	w	w/o	w	w/o	w	w/o	w	w/o	w	w/o
1)	UFF	0.10	0.06	0.26	0.16	0.11	0.07	0.29	0.17	0.09	0.08	0.23	0.21
	MMFF	0.07	0.05	0.19	0.13	0.08	0.05	0.21	0.14	0.09	0.08	0.26	0.22
	xTB	0.04	0.02	0.09	0.06	0.19	0.15	0.22	0.13	0.09	0.08	0.28	0.22
	PM6	0.06	0.04	0.15	0.09	0.06	0.05	0.14	0.09	0.12	0.13	0.38	0.29
2)	ETKDG	0.35	0.17	0.92	0.54	0.35	0.15	0.93	0.44	0.37	0.24	0.94	0.70
	ETKDG UFF	0.32	0.14	0.90	0.50	0.33	0.12	0.87	0.40	0.36	0.23	0.90	0.69
	ETKDG MMFF	0.31	0.13	0.90	0.49	0.32	0.11	0.84	0.39	0.37	0.24	0.93	0.69
3)	Gen3D	0.32	0.14	0.85	0.50	0.34	0.13	0.79	0.42	0.35	0.22	0.88	0.66
	Gen3D MMFF	0.32	0.14	0.85	0.50	0.34	0.13	0.79	0.42	0.35	0.22	0.88	0.66
4)	Null		0.84				0.88				0.77		
	Bond Order	0.41	0.17	0.98	0.48	0.51	0.24	1.02	0.60	0.31	0.22	0.76	0.51
	Bond Hop	0.38	0.13	0.85	0.44	0.41	0.14	0.90	0.43	0.32	0.23	0.77	0.54
	Bond Length	0.38	0.13	0.87	0.46	0.41	0.12	0.91	0.42	0.26	0.21	0.69	0.41
	CM	0.42	0.16	0.91	0.50	0.46	0.17	0.98	0.53	0.28	0.24	0.70	0.42
	BoB	0.40	0.16	0.94	0.48	0.45	0.15	0.96	0.52	0.27	0.23	0.70	0.42
5)	Null		0.17				0.17				0.17		
	Bond Length		0.06				0.06				0.05		

III. SUPPLEMENTARY FIGURES

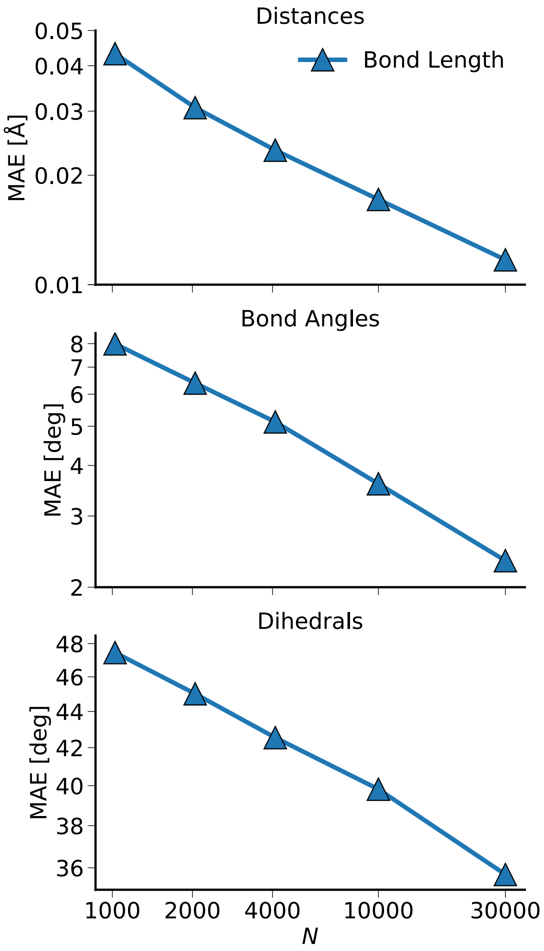

Supplementary Figure 1. Systematic improvement of prediction accuracy of the Z-Matrix components atom distances, bond angles and dihedral angles for QM9 $C_7O_2H_{11}$ constitutional isomer set using the bond length representation.

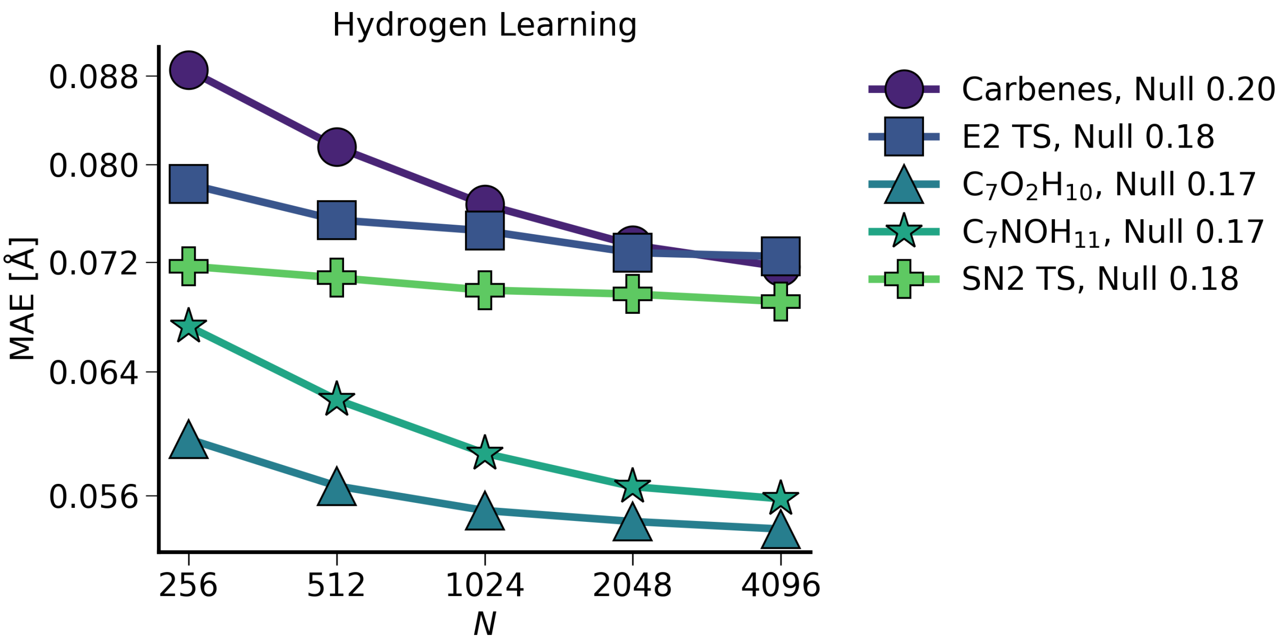

Supplementary Figure 2. Learning curves showing the MAE of pairwise distances of hydrogens to the closest four heavy atom neighbors. The null model represents the baseline accuracy calculated using the average pairwise distances in a dataset as a predictor.

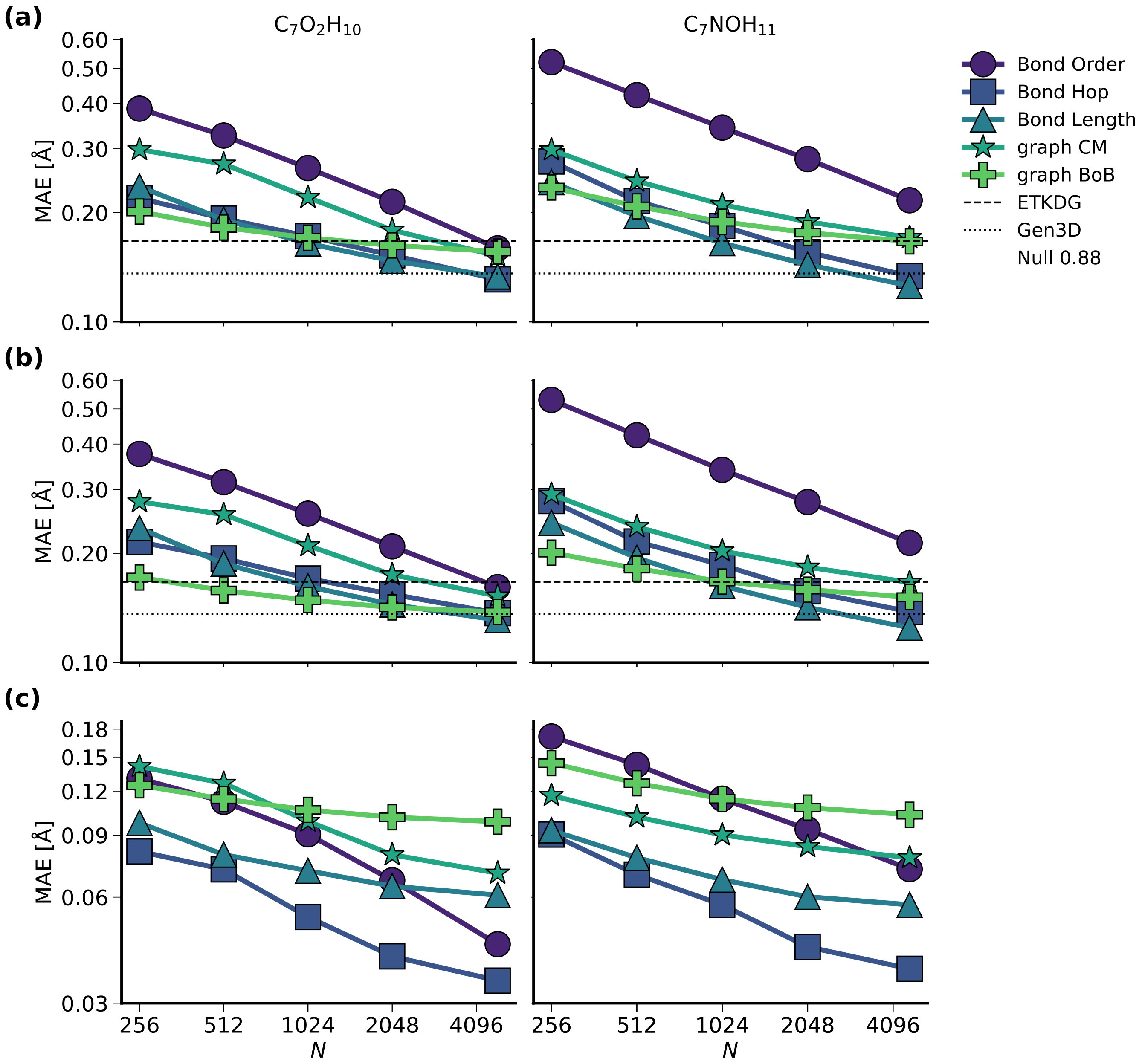

Supplementary Figure 3. Learning curves of the QM9 constitutional isomers showing the MAE of pairwise distances of heavy atoms with increasing training set sizes N. The null model represents the baseline accuracy calculated using the average pairwise distances in a dataset as a predictor. (a) MAE before 3D reconstruction. (b) MAE after 3D reconstruction. (c) MAE between the distances before and after reconstruction.

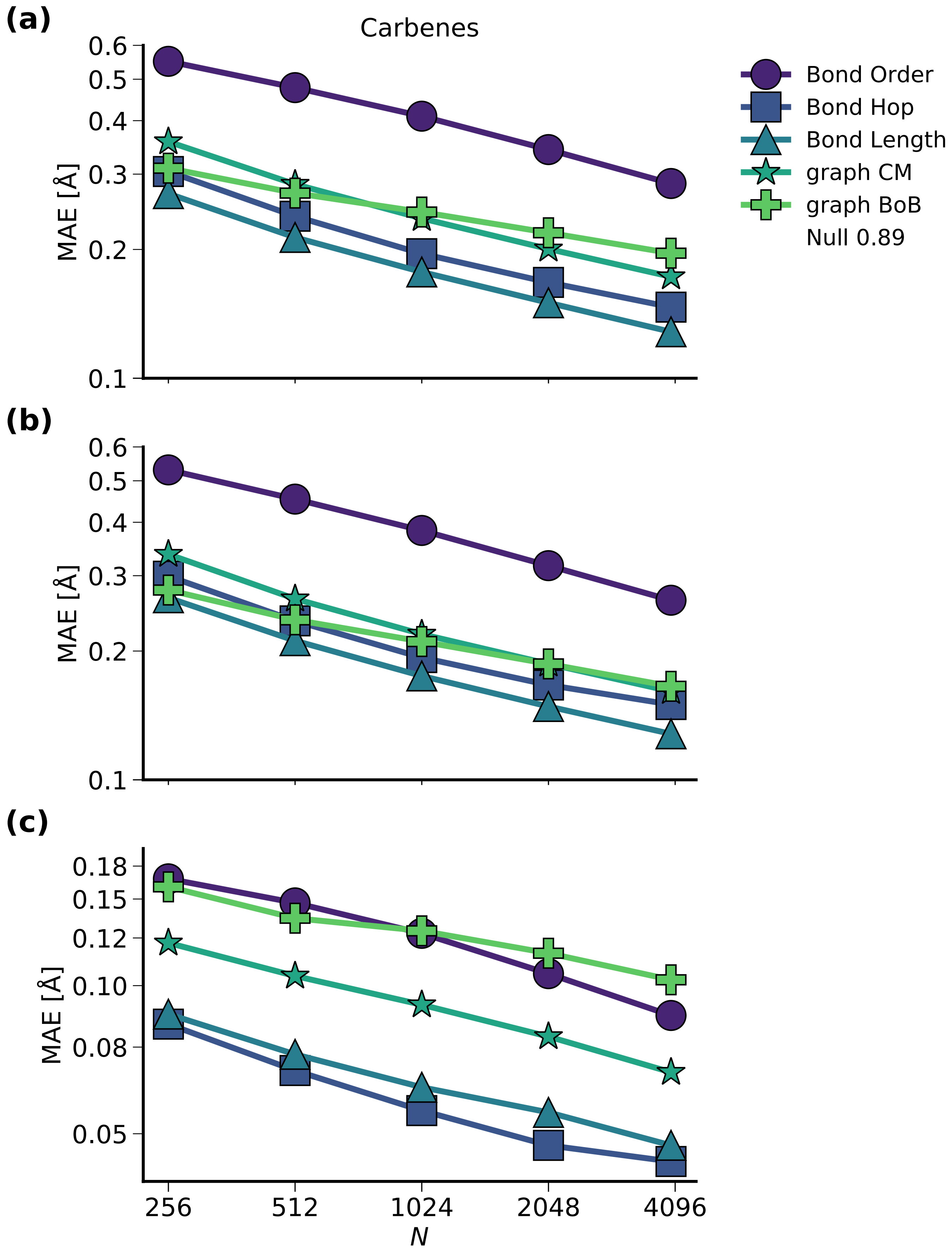

Supplementary Figure 4. Learning curves of the QMSpin carbene dataset showing the MAE of pairwise distances of heavy atoms with increasing training set sizes N. The null model represents the baseline accuracy calculated using the average pairwise distances in a dataset as a predictor. (a) MAE before 3D reconstruction. (b) MAE after 3D reconstruction. (c) MAE between the distances before and after reconstruction.

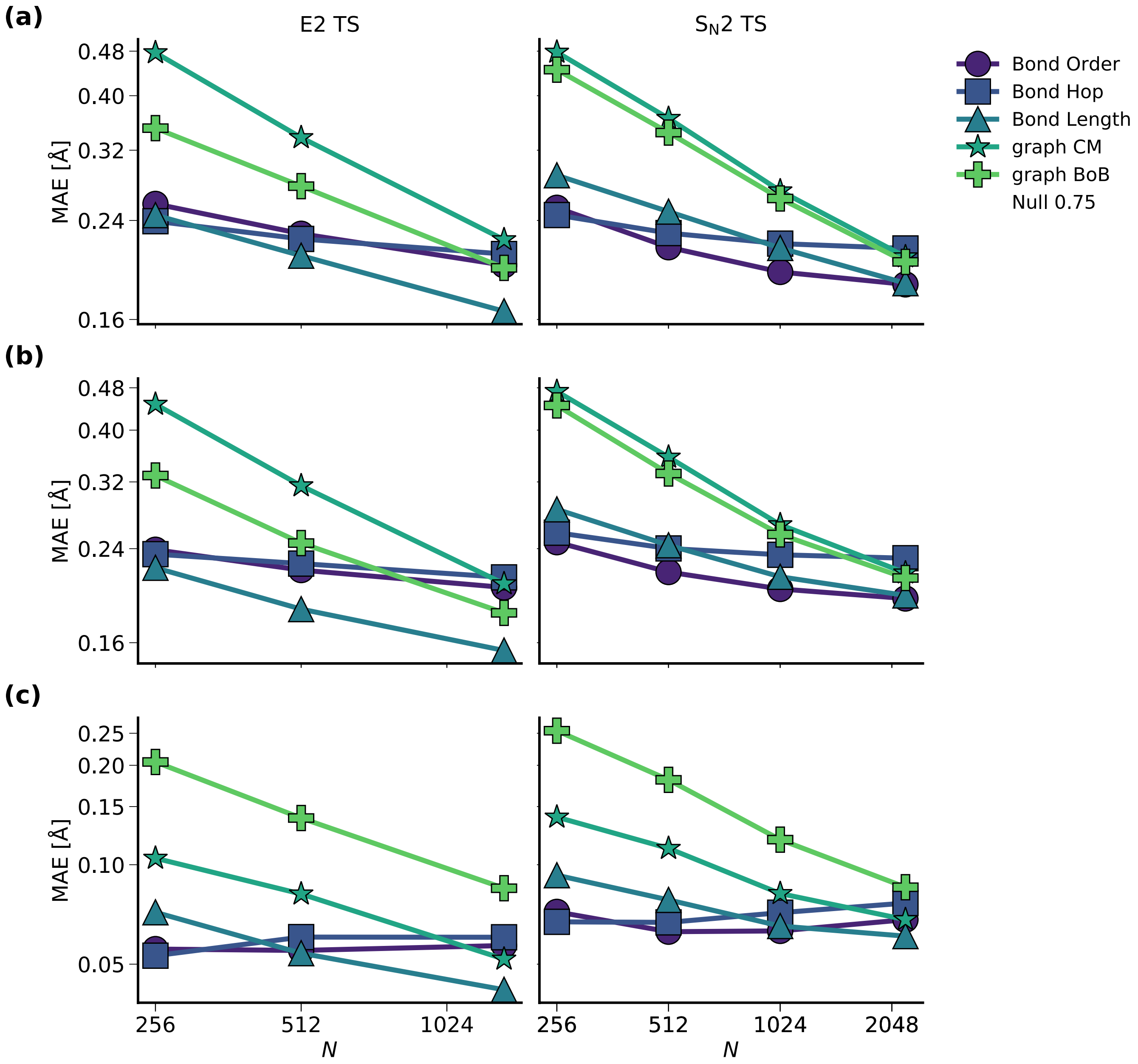

Supplementary Figure 5. Learning curves of QMrxn20 E2/S_N2 transition states showing the MAE of pairwise distances of heavy atoms with increasing training set sizes N. The null model represents the baseline accuracy calculated using the average pairwise distances in a dataset as a predictor. (a) MAE before 3D reconstruction. (b) MAE after 3D reconstruction. (c) MAE between the distances before and after reconstruction.

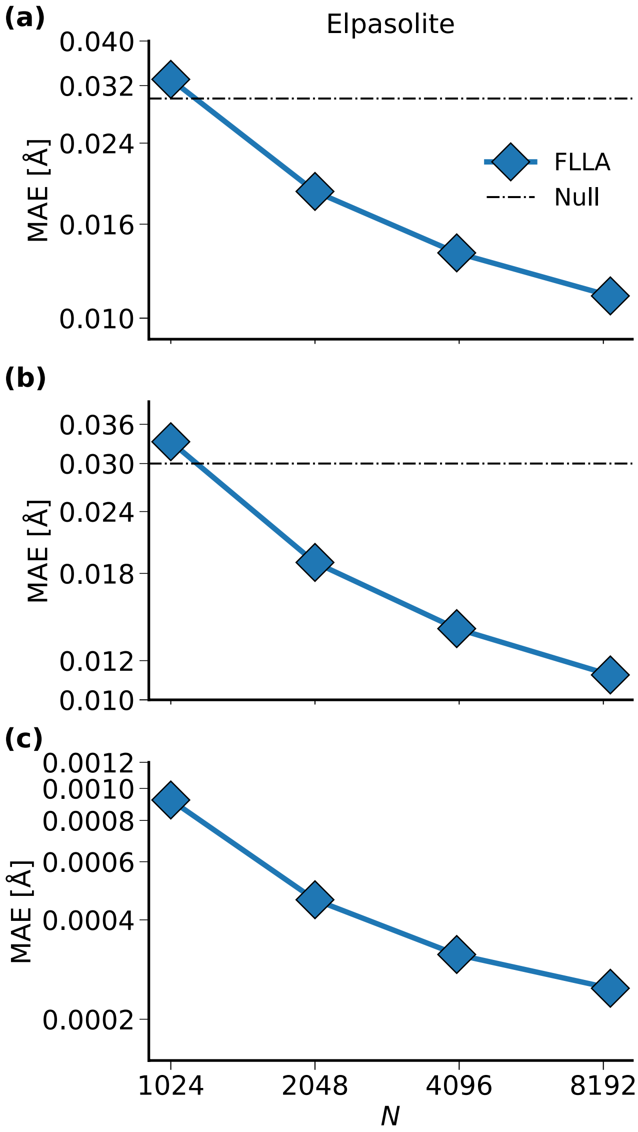

Supplementary Figure 6. Learning curves of elpasolite crystals showing the MAE of pairwise fractional distances of atoms with increasing training set sizes N. The null model represents the baseline accuracy calculated using the average pairwise distances in a dataset as a predictor. (a) MAE before 3D reconstruction. (b) MAE after 3D reconstruction. (c) MAE between the distances before and after reconstruction.

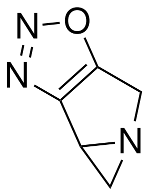

qm9_unchar_33

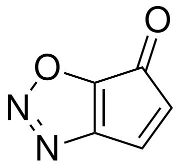

qm9_unchar_62

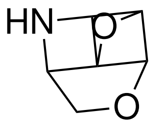

qm9_unchar_67

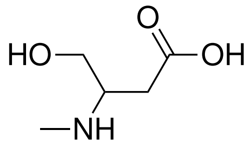

qm9_unchar_68

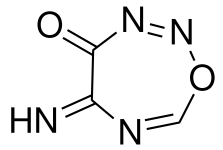

qm9_unchar_105

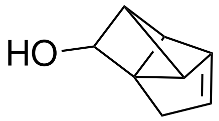

qm9_unchar_139

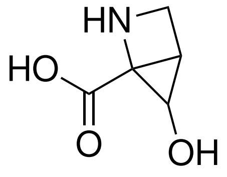

qm9_unchar_169

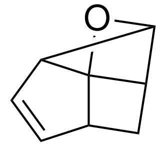

qm9_unchar_172

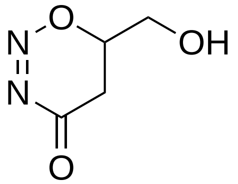

qm9_unchar_173

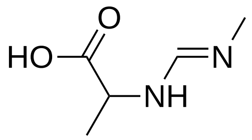

qm9_unchar_174

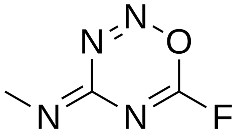

qm9_unchar_175

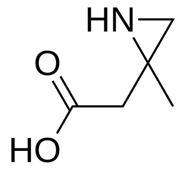

qm9_unchar_176

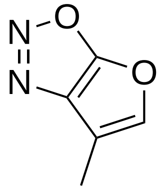

qm9_unchar_194

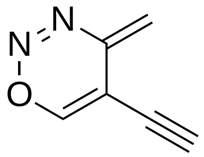

qm9_unchar_195

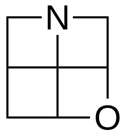

qm9_unchar_207

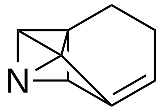

qm9_unchar_268

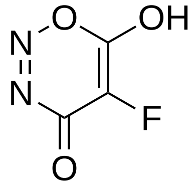

qm9_unchar_269

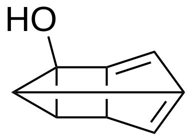

qm9_unchar_270

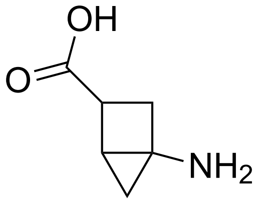

qm9_unchar_319

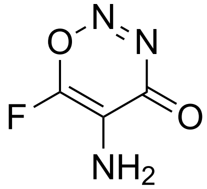

qm9_unchar_330

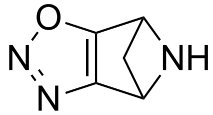

qm9_unchar_331

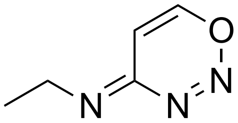

qm9_unchar_332

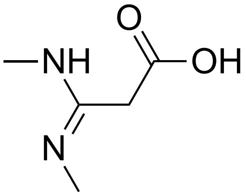

qm9_unchar_333

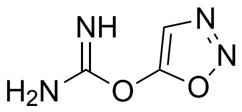

qm9_unchar_334

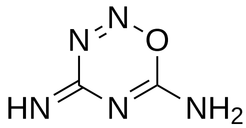

qm9_unchar_337

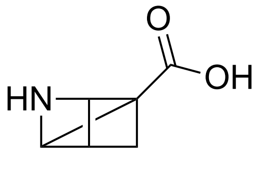

qm9_unchar_340

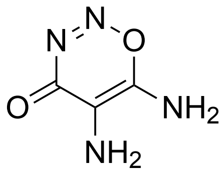

qm9_unchar_343

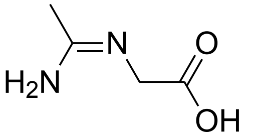

qm9_unchar_345

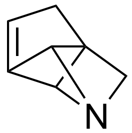

qm9_unchar_346

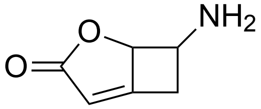

qm9_unchar_357

Supplementary Figure 7. 30 exemplary 2D structures of uncharacterized QM9 molecules which after structure generation with G2S that dissociated during geometry optimization at B3LYP/6-31G(2df,p) level of theory.

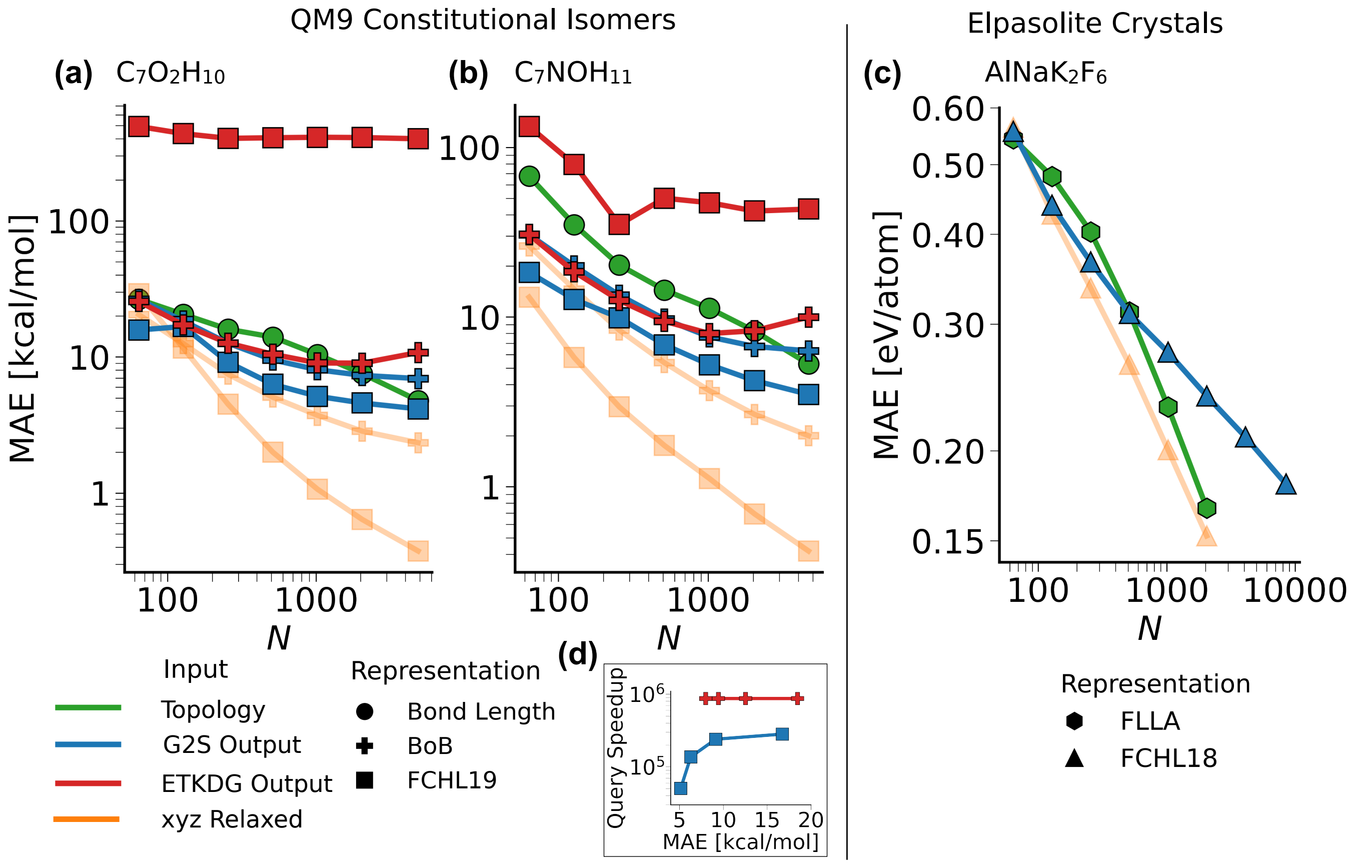

Supplementary Figure 8. Systematic improvement of energy prediction accuracy with increasing training data using G2S predictions (blue) as well as DFT structures (orange)and ETKDG/UFF structures (red) as an input to QML models. (a) and (b) atomization energy prediction of $C_7O_2H_{10}$ and C_7NOH_{11} constitutional isomers, respectively. (c) Prediction of formation energies of elpasolite crystals. (d) Speedup estimate of a G2S (blue) or ETKDG/UFF (red) based QML model over a DFT dependent QML model. This assumes an average of 16 DFT optimization steps required before a structure can be used in QML.

IV. SUPPLEMENTARY REFERENCES

[1]C. R. Harris, K. J. Millman, S. J. van der Walt, R. Gommers, P. Virtanen, D. Cournapeau, E. Wieser, J. Taylor, S. Berg, N. J. Smith, R. Kern, M. Picus, S. Hoyer, M. H. van Kerkwijk, M. Brett, A. Haldane, J. F. del Río, M. Wiebe, P. Peterson, P. Gérard-Marchant, K. Sheppard, T. Reddy, W. Weckesser, H. Abbasi, C. Gohlke, and T. E. Oliphant, “Array programming with NumPy,” *Nature*, vol. 585, pp. 357–362, Sept. 2020. Number: 7825 Publisher: Nature Publishing Group.

[2]P. Virtanen, R. Gommers, T. E. Oliphant, M. Haberland, T. Reddy, D. Cournapeau, E. Burovski, P. Peterson, W. Weckesser, J. Bright, S. J. van der Walt, M. Brett, J. Wilson, K. J. Millman, N. Mayorov, A. R. J. Nelson, E. Jones, R. Kern, E. Larson, C. J. Carey, I. Polat, Y. Feng, E. W. Moore, J. VanderPlas, D. Laxalde, J. Perktold, R. Cimrman, I. Henriksen, E. A. Quintero, C. R. Harris, A. M. Archibald, A. H. Ribeiro, F. Pedregosa, P. van Mulbregt, and SciPy 1.0 Contributors, “SciPy 1.0: Fundamental Algorithms for Scientific Computing in Python,” *Nature Methods*, vol. 17, pp. 261–272, 2020.

[3]N. Schlömer, N. Papior, D. Arnold, M. Ancellin, and R. Zetter, “nschloe/quadpy v0.16.5,” Dec. 2020.

[4]“RDKit: Open-source cheminformatics http://www.rdkit.org.”

[5]G. Csardi and T. Nepusz, “The igraph software package for complex network research,” *InterJournal*, vol. Complex Systems, p. 1695, 2006.

[6]J. C. Kromann and J. H. Jensen, “Convert cartesian coordinates to one or more molecular graphs,” *Github*, 2021. https://github.com/jensengroup/xyz2mol.

[7]Y. Kim and W. Y. Kim, “Universal structure conversion method for organic molecules: From atomic connectivity to three-dimensional geometry,” *Bulletin of the Korean Chemical Society*, vol. 36, pp. 1769–1777, June 2015.

[8]A. S. Christensen, F. A. Faber, B. Huang, L. A. Bratholm, A. Tkatchenko, K.-R. Müller, and O. A. v. Lilienfeld, “"QML: A Python Toolkit for Quantum Machine Learning" https://github.com/qmlcode/qml,” 2017.

[9]J. D. Hunter, “Matplotlib: A 2D Graphics Environment,” *Computing in Science Engineering*, vol. 9, pp. 90–95, May 2007. Conference Name: Computing in Science Engineering.

[10]M. Waskom and t. s. d. team, “mwaskom/seaborn,” Sept. 2020.

[11]W. Humphrey, A. Dalke, and K. Schulten, “VMD: Visual molecular dynamics,” *Journal of Molecular Graphics*, vol. 14, pp. 33–38, Feb. 1996.
